# Fidelity of 3D Printed Brains from MRI Scan in Children with Pathology (Prior Hypoxic Ischemic Injury)

**DOI:** 10.1007/s10278-022-00723-7

**Published:** 2022-10-24

**Authors:** Anith Chacko, Phassawan Rungsiprakarn, Ivan Erlic, Ngoc Jade Thai, Savvas Andronikou

**Affiliations:** 1 School of Clinical Sciences, Faculty of Medicine, University of Bristol, Bristol, BS28DX UK; 2 Department of Radiology, Children’s Hospital of Philadelphia, Philadelphia, PA USA; 3 Department of Radiology, Perelman School of Medicine, University of Pennsylvania, Philadelphia, PA USA; 4 Undergraduate in the Department of Mathematics, University College London, London, UK

**Keywords:** 3D printing, Pediatric brain, Cerebral palsy, Fidelity, Hypoxic ischemic injury, Magnetic resonance imaging

## Abstract

Cortical injury on the surface of the brain in children with hypoxic ischemic injury (HII) can be difficult to demonstrate to non-radiologists and lay people using brain images alone. Three-dimensional (3D) printing is helpful to communicate the volume loss and pathology due to HII in children’s brains. 3D printed models represent the brain to scale and can be held up against models of normal brains for appreciation of volume loss. If 3D printed brains are to be used for formal communication, e.g., with medical colleagues or in court, they should have high fidelity of reproduction of the actual size of patients’ brains. Here, we evaluate the size fidelity of 3D printed models from MRI scans of the brain, in children with prior HII. Twelve 3D prints of the brain were created from MRI scans of children with HII and selected to represent a variety of cortical pathologies. Specific predetermined measures of the 3D prints were made and compared to measures in matched planes on MRI. Fronto-occipital length (FOL) and bi-temporal/bi-parietal diameters (BTD/BPD) demonstrated high interclass correlations (ICC). Correlations were moderate to weak for hemispheric height, temporal height, and pons-cerebellar thickness. The average standard error of measurement (SEM) was 0.48 cm. Our results demonstrate high correlations in overall measurements of each 3D printed model derived from brain MRI scans versus the original MRI, evidenced by high ICC values for FOL and BTD/BPD. Measures with low correlation values can be explained by variability in matching the plane of measurement to the MRI slice orientation.

## Introduction

Fifteen to 28% of children with cerebral palsy have the disorder as a result of neonatal hypoxic-ischemic injury (HII) to the brain [1, 2]. Perinatal HII is most often bilateral and symmetric and it can be challenging for inexperienced radiologists to distinguish normal neonatal brains from pathologic brains on diagnostic imaging [3]. It is particularly difficult to demonstrate bilateral, symmetric cortical watershed injury in children with HII on magnetic resonance imaging (MRI) scans to non-radiologists and non-medical personnel (e.g., lay people such as parents) [4]. With advances in three-dimensional (3D) printing, we can now create palpable 3D objects based on diagnostic imaging, including MRI. One advantage of 3D prints over the diagnostic images when maintaining a 1:1 scale in the prints is an appreciation of brain volume. Another is the appreciation of bilateral cortical surface abnormality of the brain, such as that seen from delayed MRI scans in watershed injury. 3D prints of pediatric brain MRI scans have already been created in the setting of HII. These have accurately demonstrated regional cortical volume loss and altered cortical structure related to partial-prolonged type HII, as well as represented the brain size when compared against examples of age-matched normal brains [5]. These 3D models can be a helpful aid for medical training, presurgical assessment, and importantly in communicating findings in clinical consultations, to patients and parents of patients as well as for medical courtroom evidence. The accuracy and lifelikeness of these models allow lawyers, judges, jury members, medical trainees, physicians, parents, and patients to physically hold pathologic brains in order to appreciate the nature and severity of the insult [5, 6].

Pediatric brains with cortical atrophy present challenges for 3D printing of high fidelity models [5, 7]. If comparison of 1:1 scale 3D prints is to be made against normal age matched 3D printed brains, or procedural planning is to be accurate, then fidelity in the size of the pathologic printed brains needs to be maintained. Multiple authors have noted this, concluding that high fidelity is crucial for 3D printed models to be helpful in preoperative consultation, surgical planning, and resident education [8–10].

A literature review of clinical 3D printing in reconstructive surgery demonstrates that 3D printing from conventional multidetector CT has acceptable accuracy for clinical application. However, errors during image segmentation have been reported with some systems, e.g., the Peninsula 3D printing technique [11]. Moreover, and more importantly when 3D printing from MRI scans (which involves multiple steps converting to a variety of formats, skull stripping, and segmentation prior to actual printing), each step of printing 3D medical models can affect model accuracy and reproducibility [12]. Chang et al. reported that midface printed models may be more prone to error than those of other craniofacial regions because of the presence of thin walls and small projections [13]. Kondo et al. concluded that reproducibility of microsurgical anatomy of skull bone and main arteries was favorable, and that vascular lengths were also molded with high-level accuracy but that the thickness of arteries and size of cerebral aneurysms may have potential of error [14]. Another paper described successful high fidelity 3D printed personalized models of pediatric cerebrovascular diseases [15]. Several studies have evaluated accuracy of 3D printings in surgical reconstruction [16–21] and dental casting 3D printing [22]. However, other than one paper by Andronikou et al. [5] which evaluated the diagnostic reproduction of pathology on 3D prints in children with HII, there is no publication describing objective fidelity testing of 3D prints of pediatric brains. Importantly, there has been no testing of the size fidelity of pathologic pediatric brain 3D printing from MRI.

Therefore, we aimed to evaluate the size fidelity of 3D printed models from MRI scans of the brain, in children with prior HII.

## Materials and Methods

### Inclusion Criteria

Twelve MRI scans were systematically selected from the most recent studies in an MRI database of children presenting with HII for whom consent for participation could be obtained. The database contains over 2000 MRI brain scans of children presenting with cerebral palsy. Of these, 253 cases were randomly chosen for reading by 3 radiologists to determine the pathology underlying the presentation. Thereafter, 40 patients were selected for segmentation and potential printing of 3D models. However, only 12 cases eventually had 3D printed models created due to limitations with printer availability and costs. Those patients in whom delayed MRI scans (ideal for demonstrating cortical/surface atrophy) and 3D T1W sequences were available were included.

### Exclusion Criteria

Studies with severe motion artifacts on MRI were excluded. Studies with severe diffuse multicystic encephalomalacia or severe hydrocephalus were also excluded because the resultant extreme cortical thinning made automatic segmentation almost impossible and because printers available to us at the time did not have the spatial resolution to print such thin layers of cortex.

**Fig. 1:**
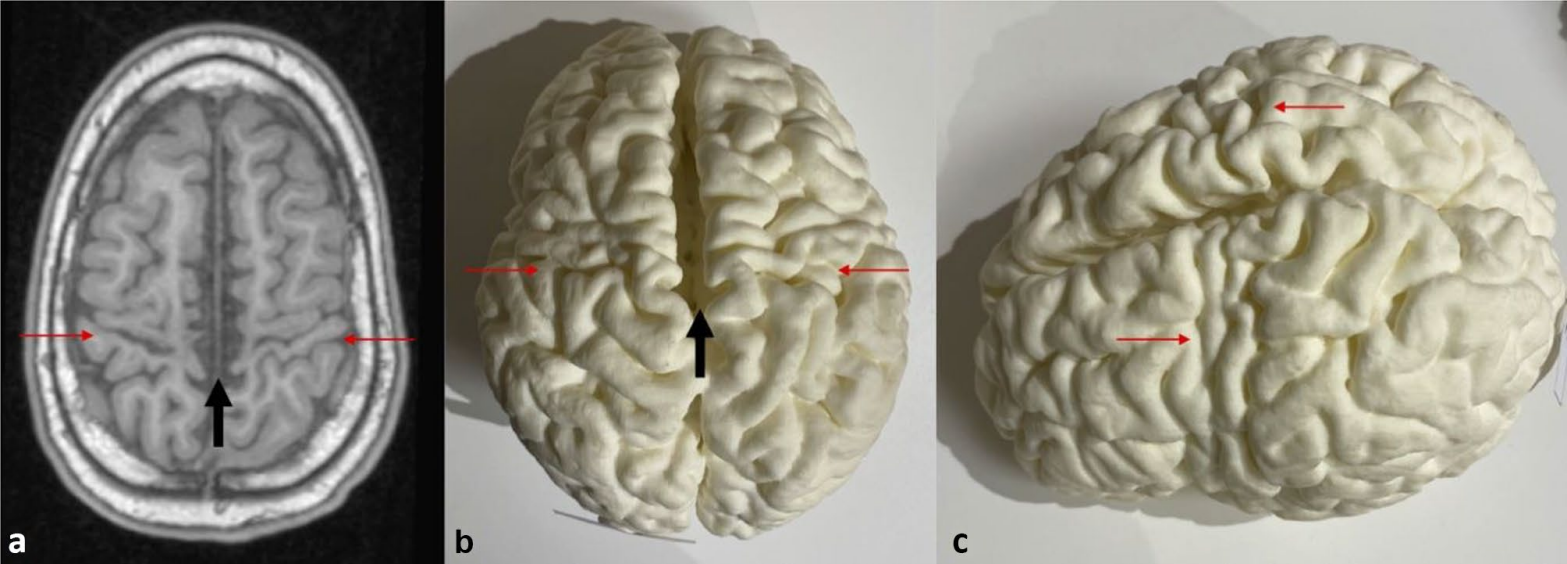
Comparison of axial T1W MRI image (**a**) with vertex view (**b**) and side oblique view (**c**) of the corresponding 3D printed model. The areas depicted by the red arrows correspond to subtle localized atrophy involving the peri-rolandic region of both cerebral hemispheres, sustained from a remote prior profound hypoxic ischemic injury in an 11-year 5-month-old girl. The black arrows depict the inter-hemispheric/lentiform separation due to the atrophy. The MRI image (**a**) depicts one aspect of the atrophy while the 3D printed model image (**c**) demonstrates the extension of involvement to the inferior and lateral aspects of the left pre-central gyrus to good effect

**Fig. 2:**
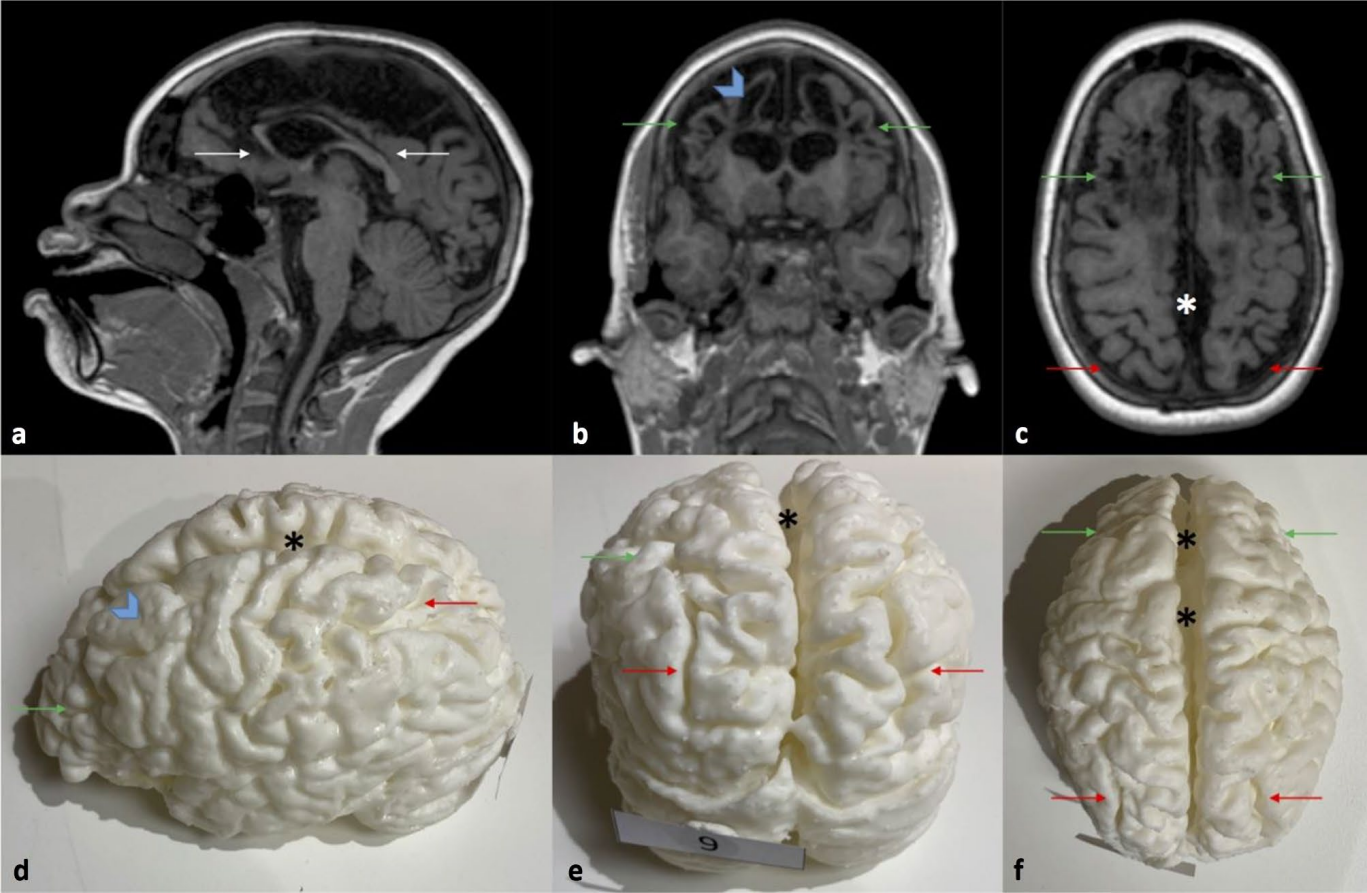
T1W MRI images in sagittal (**a**), coronal (**b**), and axial (**c**) planes with images of the corresponding 3D printed model in different views (**d**–**f**). This is a 10-year 6-month-old girl who suffered a remote prior hypoxic ischemic injury at term, with a combined partial prolonged and profound injury. The corpus callosum is diffusely thinned as a result of global atrophy (white arrows in (**a**)). The green arrows depict areas of focal cystic encephalomalacia and ulegyria within the anterior inter-arterial watershed of both cerebral hemispheres and the corresponding overlying cortical atrophy. The blue chevron depicts an area that required a manual patch (after automatic segmentation) to cover a defect due to the segmentation not recognizing the residual thin cortex overlying the cystic encephalomalacia. The red arrows depict atrophy with deep sulci of the posterior inter-arterial watershed bilateral. The asterisks (white and black) depict inter-hemispheric fissure widening due to the diffuse atrophy and associated lentiform separation of the hemispheres at the involved regions

As mentioned, only 12 patients were selected for 3D printing due to limitations of printer availability and cost. Of these, 3 demonstrated the basal ganglia and thalamic (BGT) pattern with perirolandic cortical atrophy, 3 had focal cystic encephalomalacia, 3 had cortical watershed atrophy, 2 had diffuse cortical atrophy, and 1 was normal. Figures 1 and 2 show examples of the corresponding MRIs and 3D printed models.

### 3D Printing Technique

Segmentation of the 3DT1 sequence involved several processing steps. DICOM (Digital Imaging and Communications in Medicine) data were converted into a NIfTI file. The NIfTI file was then “segmented” using SPM12 (Statistical Parametric Mapping, The Wellcome Center for Human Neuroimaging, UCL Queen Square Institute of Neurology, London), in MATLAB (MathWorks, Natick, MA, USA) where each voxel was assigned to one of several tissue classes depending on its intensity, and voxels corresponding to the skull were “stripped.” The files containing the tissue types that composed the solid structure of the brain (grey and white matter) were then combined to create a new volume representing the structure of the brain, excluding the cerebrospinal fluid. From this, a surface was approximated in stereolithography (STL) format. For the purposes of defining the brain surface, internal volume data was discarded leaving only the outer boundary. STL surface files also referred to as “meshes,” are a commonly used 3D printing format that builds a surface of triangles, each defined by a set of 3 coordinates and an outward facing normal vector. The generated STL surface usually contains meshing errors which are anomalies in the file that can cause printing failures. We made several mesh repairs to prepare files for printing. Repairs were made using MRIcros or isosurface, both MATLAB applications. Then the STL file was “sliced” into a set of printable layers. The fused deposition modeling (FDM) printing method was used. This is a material extrusion type of 3D printing that is relatively low cost and widely available, and which provides good models for demonstration purposes [23].

The 3D printing machine used was a Makerbot Replicator (Makerbot Industries, Brooklyn, NY). The material used was PLA (polylactic acid) with a material thickness of 1.75 mm; however, the extruder was set to deliver the deposition thickness at 0.2 mm. The minimum layer deposition resolution achievable by the machine is 100 microns, but we set the layer height at 0.2 mm (200 microns). Previous workflows recommended 0.3 mm, but this was found to be too large and with significantly noticeable irregularity of the surface. If we went to 0.15 mm, the finish would be much smoother but the print time would be more than 24 h, therefore a balance between print time and finish quality was considered optimal.

### Linear Measurements on Pediatric Brain MRI Scans

**Fig. 3:**
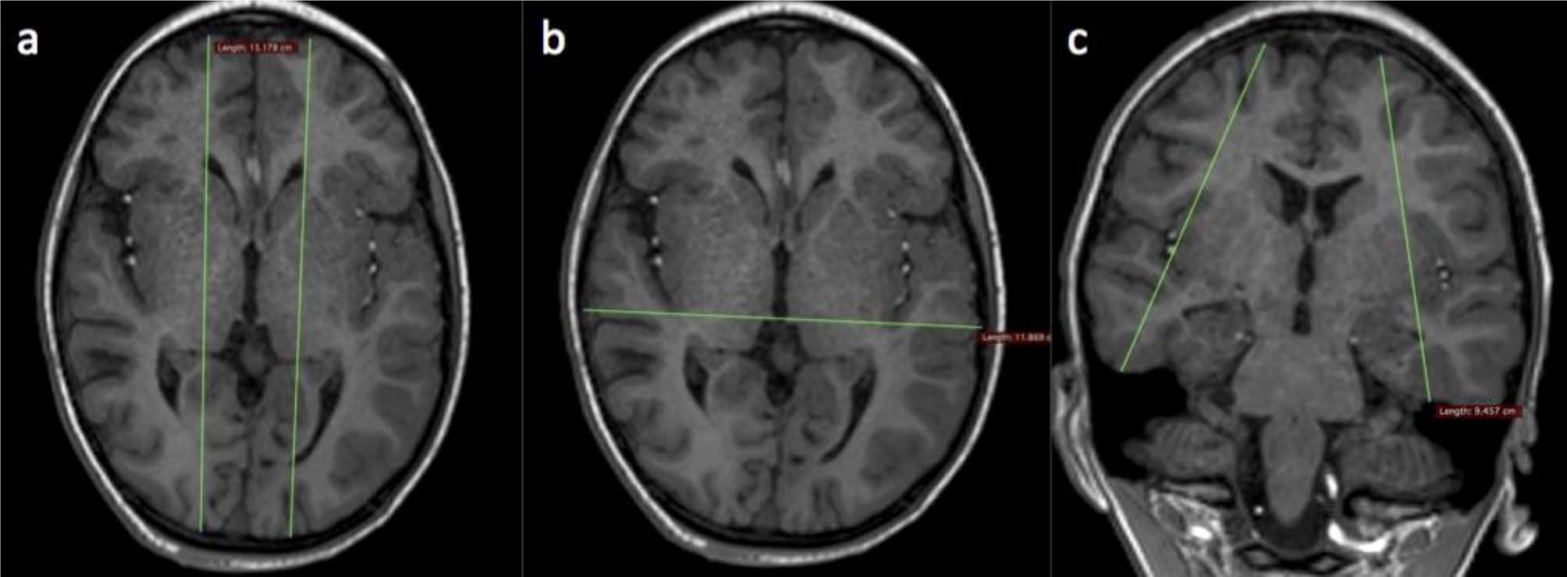
Linear measures on T1W MRI images. Fronto-occipital measures in the axial plane (**a**). Bi-temporal or bi-parietal diameter in the axial plane (**b**). Hemispheric height in the coronal plane (**c**)

**Fig. 4:**
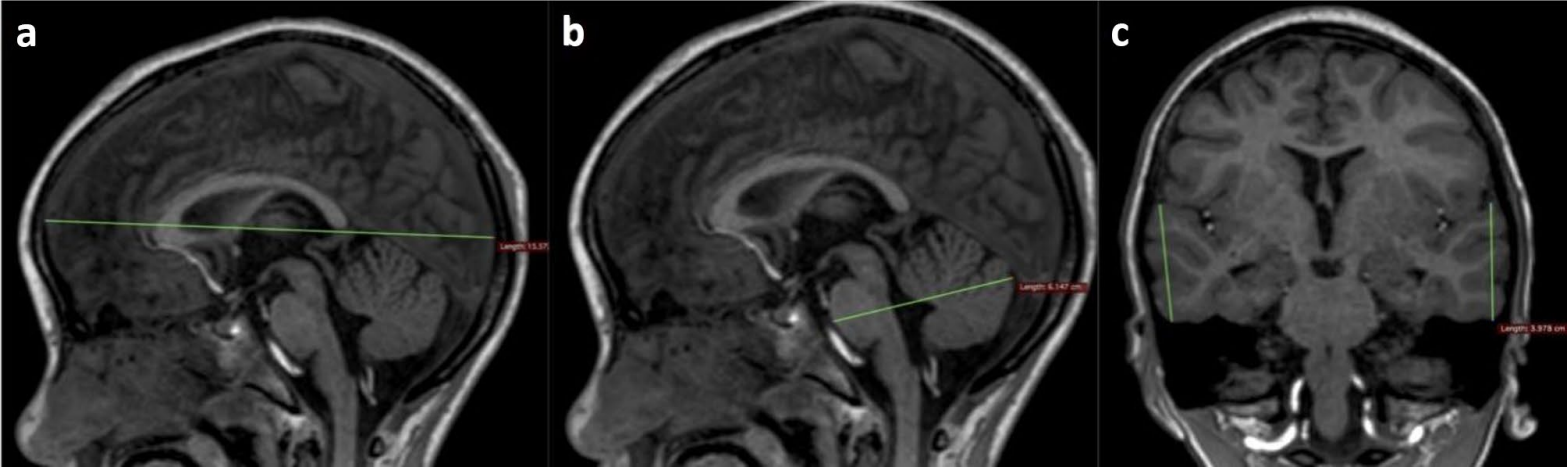
Linear measures on T1W MR images. Fronto-occipital measure on the midline sagittal image (**a**). Pons cerebellar thickness on midline sagittal image (**b**). Temporal height in the coronal plane (**c**)

Linear measurements were performed on the DICOM images of the 12 MRI scans using OsiriX (Pixmeo, Bernex, Switzerland) DICOM viewing software in various planes and at pre-determined levels/slices and recorded in centimeters (cm) as described below (Figs. 3 and 4):

1. Fronto-occipital length (FOL) in the axial plane: anterior–posterior length of each hemisphere measured on an axial view at the level of the lateral ventricle trigone (i.e., the longest part of the brain for each side).
  Definition: FOL right (Rt) and left (Lt); maximum distance between the most anterior portion of the frontal pole and the most posterior portion of the occipital pole measured parallel to the medial surface of each hemisphere.
2. Hemispheric width (HW) is measured as a bi-parietal or bi-temporal diameter (BTD/BPD) in the axial plane (i.e., the widest part of the brain).
  Definition: maximal distance perpendicular to the midline between the most lateral portions of the temporal or parietal lobes.
3. Hemispheric height (HH): in the coronal plane, superior to inferior hemispheric measurements (i.e., the tallest part of the brain).
  Definition: HH right (Rt) and left (Lt) — in the coronal plane at the level of the 3rd ventricle and foramen of Monro, the maximum height between the most superior portion of the frontal lobes to the most inferior portion of the temporal lobes, measured oblique to the interhemispheric fissure.
4. Temporal height (TH): in the coronal plane, superior to inferior measurements of the tallest part of the temporal lobes.
  Definition: TH right (Rt) and left (Lt) — in the coronal plane from the Sylvian fissure, vertical height of temporal lobes for both right (Rt) and left (Lt).
5. Fronto-occipital length midline (FOL) in the sagittal plane.
  Using the midline slice or just off the midline from the tip of frontal lobe to the tip of occiput.
6. Pons-cerebellar thickness (PCT) in the sagittal plane.
  Definition: using the midline slice — maximum anterior-posterior dimension of the pons and cerebellum from the anterior surface of the pons to the posterior part of the cerebellar vermis, perpendicular to the brain stem and cord.

### Measurements on Corresponding 3D Printed Models

**Fig. 5:**
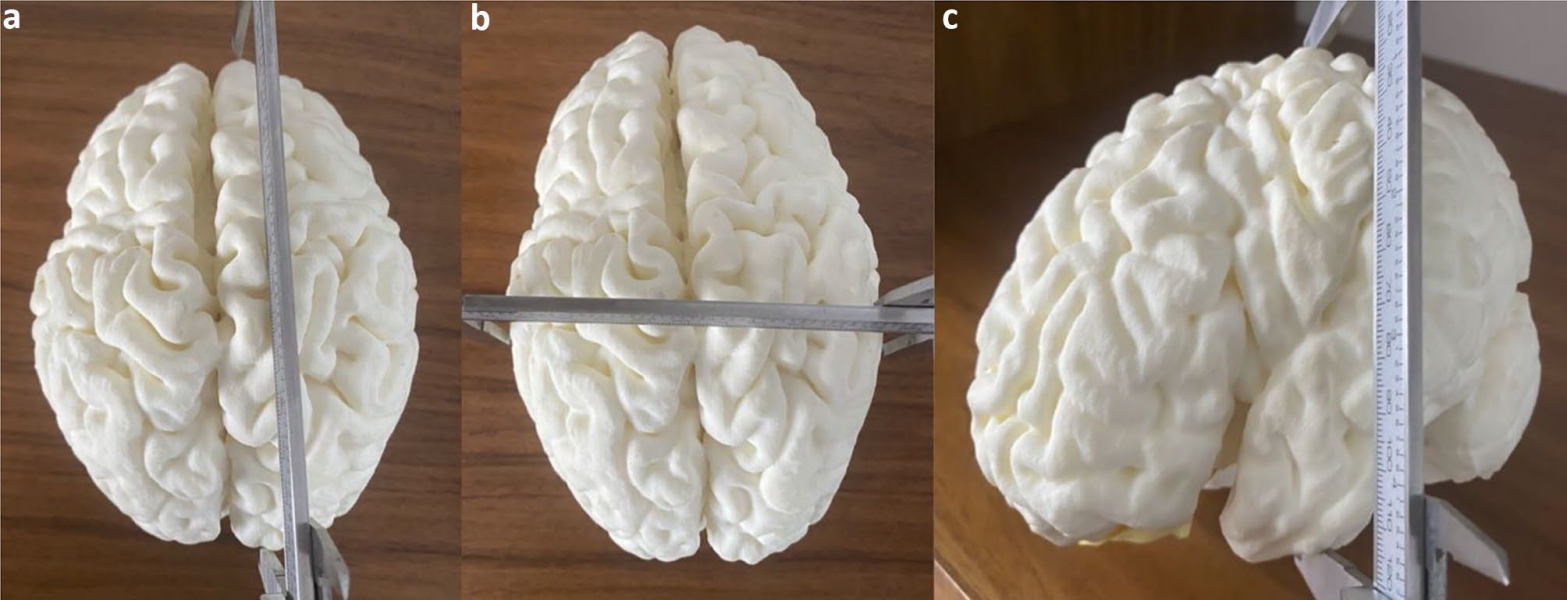
Demonstrative images of linear measures of the 3D print models using the digital Vernier calipers. Right hemispheric fronto-occipital length (**a**). Bi-parietal or bi-temporal diameter (**b**). Left hemispheric height (**c**)

One of the 12 3D printed models was excluded as a technically failed print due to material defects. The above measurements were then also made on the 3D printed models (Fig. 5) with the intention of mimicking the cross-sectional measurements. A qualified radiologist used the best approximations of corresponding measurements from the MRI scans to make the measurements on each 3D printed model using a digital Vernier caliper (Max Measure, www.maxmeasure.co.uk, UK).

Analysis of agreement was performed using interclass correlations (ICC) and standard error of measurement (SEM) with standard statistical analytic methods. ICC analyses were used to examine the similarity of the MRI versus 3D brain measures. Both ICC agreement and ICC consistency measures were examined. SEM is a function of both the standard deviation of observed scores and the reliability of the test. The higher the ICC (i.e., closer to 1) the higher the correlation. SEM is interpreted as a general measure of error between two methods that were compared and is in the same units as the measurements.

## Results

**Fig. 6:**
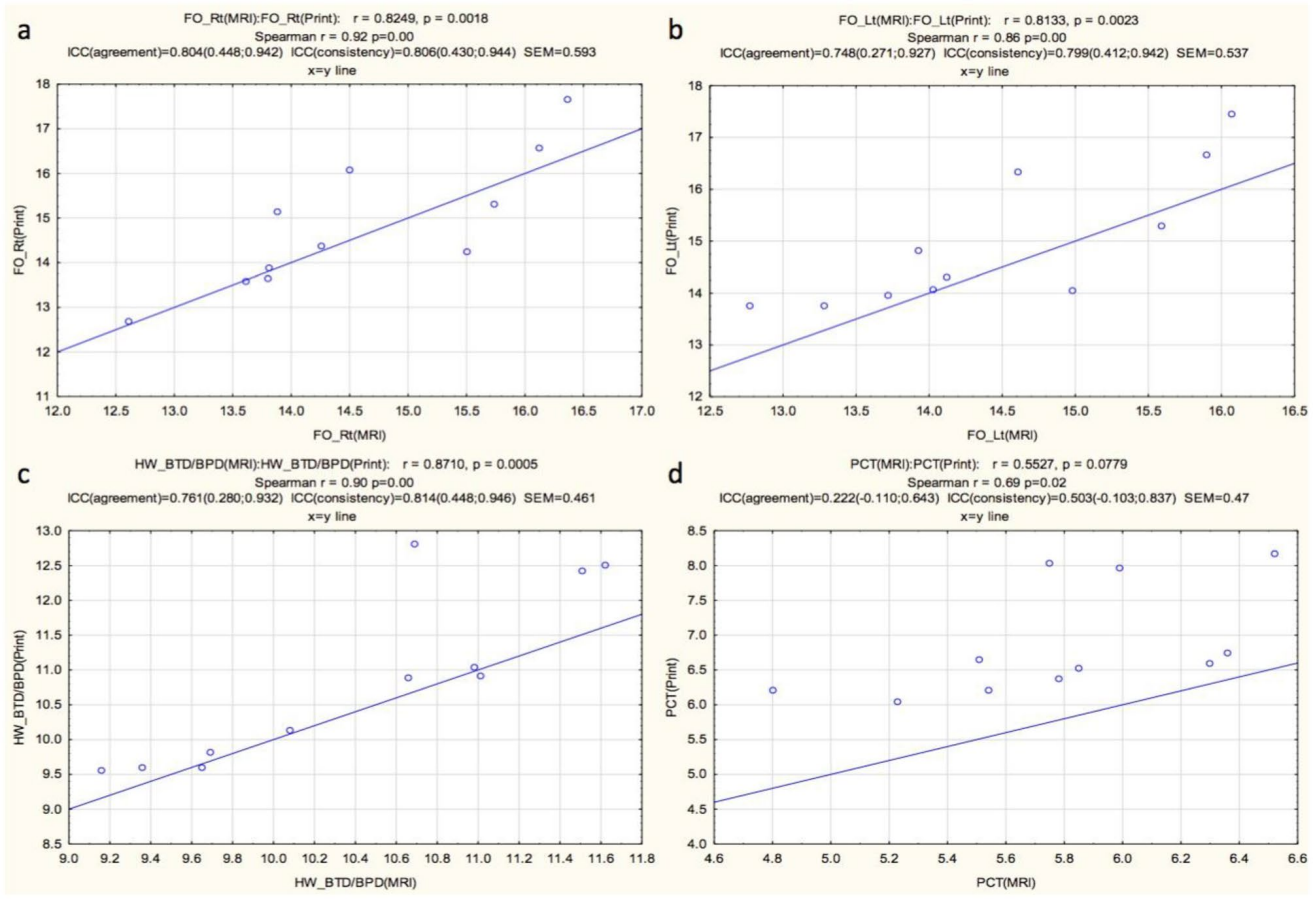
Graphic display of correlations between the MRI linear measures (MRI) and measurements obtained from the 3D print models for right fronto-occipital length (**a**), left fronto-occipital length (**b**), bi-temporal or bi-parietal diameter (**c**), and pons cerebellar thickness (**d**). (Key: r, correlation coefficient; p, statistical significance; Spearman r, Spearman’s correlation coefficient; ICC, interclass correlation coefficient; SEM, standard error of measurement). Units of SEM in centimeters (cm)

**Fig. 7:**
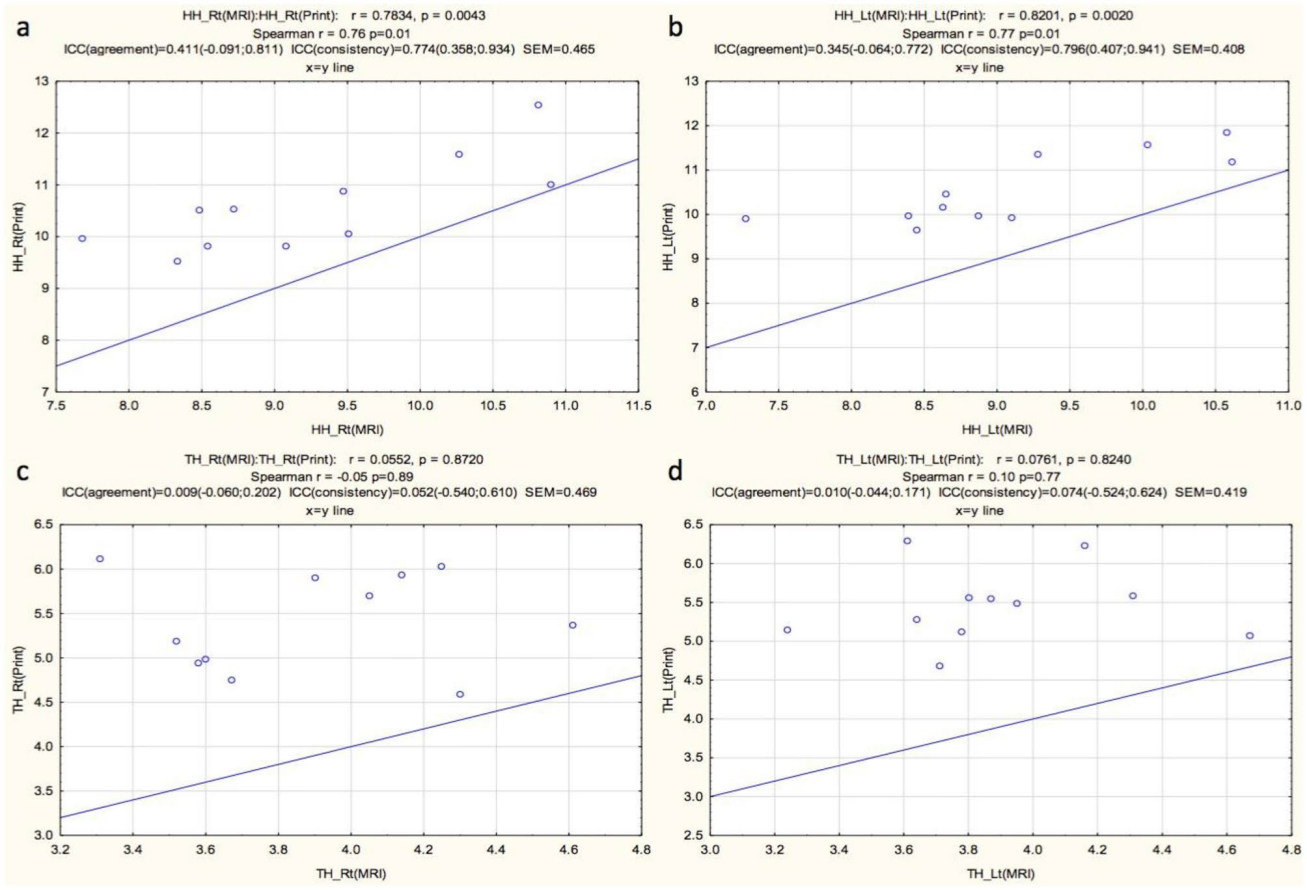
Graphic display of correlations between the MRI linear measures (MRI) and measurements obtained from the 3D print models (print) for right hemispheric height (**a**), left hemispheric height (**b**), right temporal height (**c**), and left temporal height (**d**). (Key: r, correlation coefficient; p, statistical significance; Spearman r, Spearman’s correlation coefficient; ICC (agreement), interclass correlation coefficient agreement; ICC (consistency), interclass correlation coefficient consistency; SEM, standard error of measurement). Units of SEM in centimeters (cm)

FOL and BTD/BPD fell within the range of high correlation (0.804, 0.748, and 0.761 respectively). The ICC agreement was moderate to low for the right HH, left HH, right TH, and left TH and PCT (0.411, 0.345, 0.009, 0.010, and 0.222 respectively). The ICC consistency of the right FOL, left FOL, BTD/BPD, right HH, and left HH fell within the range of high correlation (0.806, 0.799, 0.814, 0.774, and 0.796 respectively). The ICC agreement was moderate for the PCT (0.503). The ICC consistency was poor for the right TH and left TH (0.052 and 0.074 respectively). The correlations between the MRI measurements and the 3D print measurements are shown in Figs. 6 and 7 for the various regions measured. Overall measurements correlated in terms of right versus left as well as visual appreciation of general volume, for each patient. The linear measurements for the 3D prints were consistently larger than the linear measurements on the ground truth MRI scans for all measures and in all individual patients. The average standard error of measurement (SEM) was 0.48 cm and ranged from 0.408 to 0.593 cm (Table 1).

**Table 1:** Interclass correlations (ICC) of agreement and consistency, and standard error of measurement (SEM) of the various measurements for the two techniques (cross sectional MRI vs. 3D prints of the MRI)

Measurement	ICC agreement	ICC consistency	SEM (cm)
Right FOL	0.804 (0.448; 0.942)	0.806 (0.430; 0.944)	0.593
Left FOL	0.748 (0.271; 0.927)	0.799 (0.412; 0.942)	0.537
HW/BTD/BPD	0.761 (0,280; 0.932)	0.814 (0.448; 0.946)	0.461
Right HH	0.411 (− 0.091; 0.811)	0.774 (0.358; 0.934)	0.465
Left HH	0.345 (− 0.064; 0.772)	0.796 (0.407; 0.941)	0.408
Right TH	0.009 (− 0.060; 0.202)	0.052 (− 0.540; 0.610)	0.469
Left TH	0.010 (− 0.044; 0.171)	0.074 (− 0.524; 0.624)	0.419
PCT	0.222 (− 0.110; 0.643)	0.503 (− 0.103; 0.837)	0.47

*FOL* fronto-occipital length,* HW* hemispheric width,* BTD* bi-temporal diameter, *BPD* bi-parietal diameter *HH*, hemispheric height, *TH* temporal lobe height, *PCT* pons cerebellar thickness

## Discussion

Despite widespread use and growing momentum in medical applications of 3D printing, including 3D printing of children’s brains, there is surprisingly little work that proves the fidelity of 1:1 scale 3D prints with regard to measurement. This is especially true of 3D printing from MRI scans (as opposed to CT scan data) of brain structures and in children. Most importantly, there are no available data regarding reproduction of pathologic brains or those pathologies affecting complex structures such as the brain surface. Inaccuracies in reproduction of pathologic brains as 3D printed models and low fidelity of the prints can have far reaching consequences, especially if 3D prints are used for creating implants, simulation procedures, or for communication of findings that relate to size.

Our results show correlation in the overall measurements of each 3D printed brain model versus MRI ground truth data, evidenced by high ICC values for FOL (the brain in an antero-posterior dimension) and BTD/BPD (the brain in a width dimension from right to left). The lower correlation between the other measures was largely expected and probably relates to the difficulty obtaining caliper measurements in the exact same plane and position as the MRI measurements. Still, we achieved relatively good consistency, highlighting the fidelity of 1:1 scale 3D prints, particularly in patients with brain pathology. According to a publication by Andronikou et al. [5], the features of volume loss and surface abnormalities of hypoxic ischaemic injury are visible in the 3D printed model and can be used to compare with models of normal 3D printed brains side by side. Minimal expansion of the PLA material was experienced upon deposition on the model; however, this can be a factor to consider if requiring extremely accurate models, e.g., for surgical planning. For our purposes, an SEM of 4.8 mm was thought to be satisfactory as these models were for demonstration and descriptive purposes only and not to be used in surgical planning. Our results bode well for the regular use of 3D printed models to compare brains of children with and without pathology to better communicate findings.

The main limitation of measuring the 3D printed models using a caliper is that only surface measures are possible and hence were not necessarily at the exact same levels made on the cross-sectional MRI scan slices. The fidelity of measurements was adequately maintained on each model. The FO midline length, obtained on the MRI scans, was not reproducible on the 3D prints due to lack of anchor points (both anteriorly and posteriorly), and its measurement was therefore not thought reliable. Similar concerns were initially thought to be an issue with the PCT measurement; however, with this measurement, the anterior anchor was reliable at the pons and a good estimate of the posterior limit was found to be possible and reproducible.

The concern addressed in this paper was the fidelity of 1:1 scaled 3D print models to the original MRI scan data regarding size, especially considering potential use for communicating pathologic findings including in a medicolegal setting. There was also the technical concern of the fidelity of printed 3D models of abnormal/atrophic brains, as compared to the 3D printing of normal brains.

Accuracy and reproducibility metrics of 3D printing have been described in the literature but the issue is complex and dependent on multiple variables. What is acceptable reproducibility is also dependent on the intended use of the 3D printed model, with those used for implants or surgical simulation needing more stringent limits versus those used simply for communication purposes. No single variable is representative of the comprehensive accuracy of 3D printed medical models for all indications. Acceptable ranges of discrepancy between MRI or CT measurements and the associated 3D printed model can vary from 0.2 to 6 mm [24].

Almost all measurements obtained on our 3D models were greater than the corresponding MRI measurements. This was largely expected given the inherent expansion of materials during the 3D printing process. This is not considered a drawback for this particular indication of 3D printing, as cortical atrophy which is the main feature would not be artificially exaggerated through expansion of the material. Future analysis and comparison of materials and printing methods will be able to distinguish which materials are more prone to this effect and which are more accurate. For the demonstration purposes envisaged for communicating the cortical findings in HII, this size discrepancy was thought to be negligible. This is evidenced by the low standard error of measurement throughout all measurements of less than half a centimeter on average (0.48 cm or 4.8 mm).

3D printing of brains from MRI scans is established and well-documented. The barrier to entry in terms of expertise required, however, is deemed to be high. Several publications discuss workflows for 3D printing models from medical images and free online guides exist for anyone to 3D-print their own MRI scans, but in our experience, these have yielded unsatisfactory models for the abnormal pediatric MRI scans in our database [25, 26].

Especially given the wide variety of manifestations of HII and the associated structural changes to the cerebral cortex, 3D models are difficult to print in pathologic brains due to significant failure of standard automatic segmentation methods.

We, therefore, developed and refined the process and workflows involved in printing 3D models of the pathologic brains as part of the larger study in our lab (unpublished data). The 3D models were also printed using 3 different printer types to assess differences between the printers and materials. All printing methods were shown to produce high quality models of the brain with slight expected variation in quality depending on the type of printer and associated material.

A wide variety of useful information can be found regarding individual print settings for the printing step of the process. However, some of the workflows tend to prioritize speed and cost reduction over the final accuracy of models. While overall speed was a major consideration in our project due to the sample size (12 brains) and time taken to print each model, we considered surface detail the most important feature considering the nature of the models’ future use. This constraint needs to be kept in mind when printing the final models that require anatomic/pathologic accuracy. The long print times limited the number of prints available for measurement to determine fidelity of the 3D printing in our study.

There are many steps in the process of deriving a 3D printed model from an MRI scan where the size of the model may be affected in comparison to the original MRI scan.

Demonstration of cortical injuries and cerebral atrophy is critical for determining the timing, severity, and possibly the pathogenetic mechanism of the insult in pediatric HII. The specific area of concern which should be demonstrated is the watershed zone, which is preferentially injured in partial-prolonged HII [27]. However, demonstration of the pathology to the various stakeholders (predominantly lay people such as parents or patients and including lawyers and judges) on standard MR imaging is difficult due to the highly-specialized nature of the imaging as well as the need for relatively high cognitive visualization capabilities and experience, to appreciate the pathology in totality from many sequences and over multiple cross-sectional imaging slices.

Cerebral palsy can occur due to many causes such as infections, or underlying abnormalities such as metabolic and congenital disorders [28, 29], but is most often caused by perinatal HII [28]. MRI plays a significant role in diagnosis and monitoring of infants with HII. The role of MRI in this setting has been well documented [30] and the patterns of injury and timing of injury as well as correlation with clinical outcomes [31] have been investigated. Expert physicians in various fields (pediatrics, occupational therapy, pediatric radiology, etc.) are not only involved in the clinical management but are also called upon during the process of litigation to assess available information (both clinical and imaging) when there is an alleged breach in the standard of care [23, 32–34].

MRI can be used to evaluate the severity of HII and used to postulate duration and timing of the injury, especially when coupled with clinical information. For example, neuroimaging that demonstrates damage exclusively to the cerebral cortex will be suggestive of prolonged partial asphyxia. Damage shown to exclusively involve the deeper structures or structures with high metabolic rates is more suggestive of a profound or near total asphyxia. Combinations of patterns also occur. The 3D models we have produced would be most useful for demonstrating involvement at the surface of the brain, i.e., cortical watershed atrophy or localized peri-rolandic cortical atrophy. In addition, more diffuse injury causing global atrophy would also be visible from the actual size of the 3D model in comparison to an age-matched normal sized brain or when compared to a known object.

Novel methods of demonstration of the cortex have been developed with Mercator map reconstructions [35]. This has also been shown to be a valid method of demonstrating the cortical surface and associated injury to lay people [4]. 3D printed models are thought to be a more natural additional way of demonstrating pathology globally and tangibly, especially in demonstrating global injury to lay people and non-radiologist physicians.

**Fig. 8:**
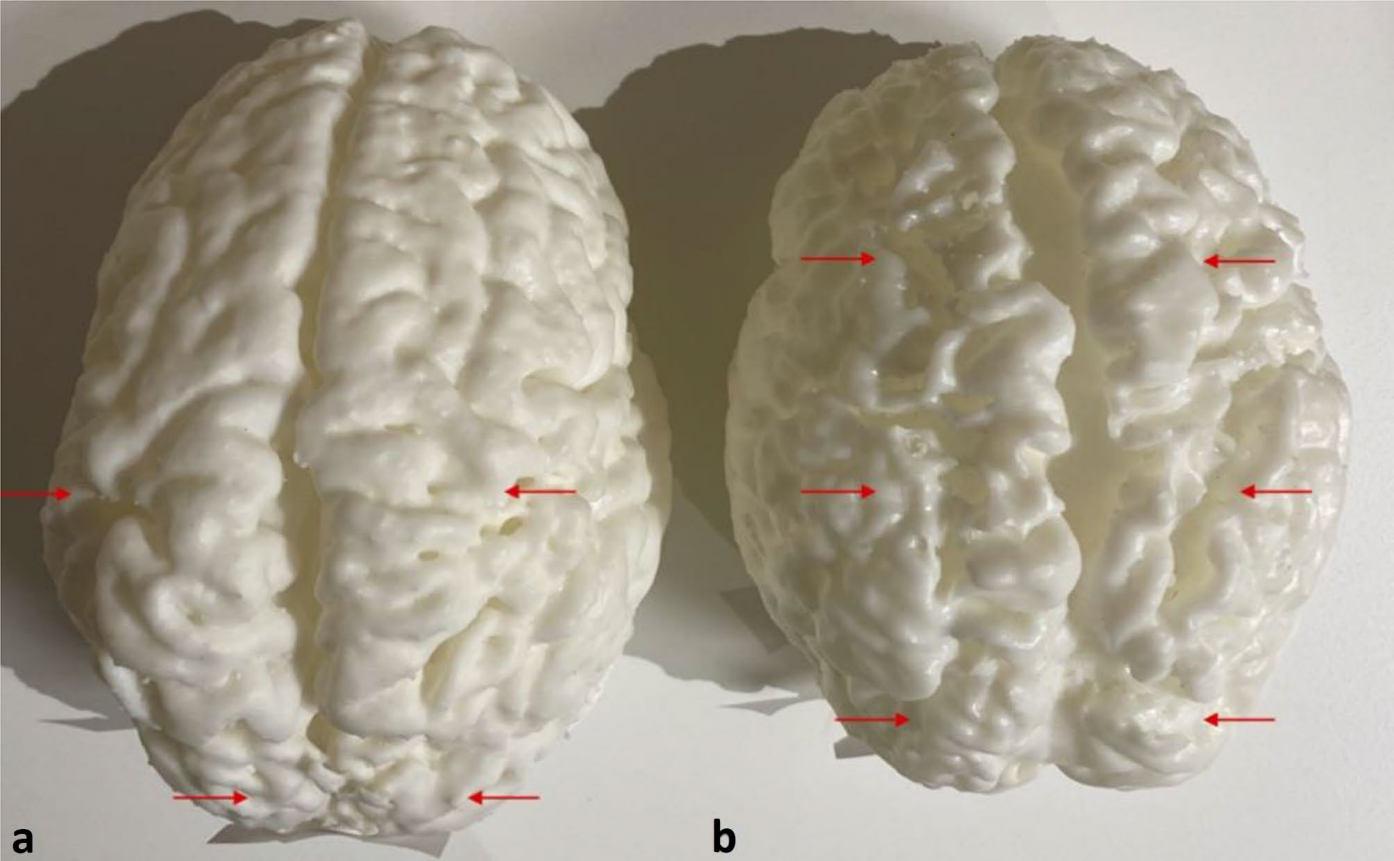
Two models of different children’s brains side by side for comparison. Both patients suffered partial prolonged hypoxic ischemic injury at term gestation, now with cerebral palsy, who had delayed MR imaging — a 2-year 2-month-old boy (**a**) and a 11-year 6-month-old boy (**b**). Note, the more severe atrophy in the 11-year-old (**b**) evident from increased depth and width of the sulci in the watershed region (red arrows in each model) as well as the low overall brain volume (i.e., an 11-year-old with a similar or smaller brain volume than a 2-year-old) when visualizing the models side by side

The advantage of obtaining 3D printed models to provide a global view of pathology and show the effect of diffuse atrophy on the brain volume is clear, as shown in Fig. 8. This is especially striking when someone is given the model to handle. In addition, when a pathologic model is placed alongside an age-matched normal model, the differences in size are immediately and easily apparent (Fig. 8). An additional advantage is the ability to view both sides simultaneously from different vantage points, and hence appreciate bilateral and symmetric disease, which defines a global injury such as the cortical changes from partial prolonged HII. Confidence in the maintenance of fidelity of linear measurements is paramount for medical practice and communicating medical findings as well as in medical litigation.

## Limitations

The study has several limitations. We did not exactly determine which portion of the processing pipeline results in measurement differences — we assume that the printing process with expansion of the printing materials is the culprit. We also assume that the MRI scan is an accurate size representation of the patient. Furthermore, we have compared external caliper measures against cross sectional slices which creates problems for measuring in the appropriate plane for some of the dimensions we devised in the coronal plane. One potential possibility is to print individual slices but this would not answer the question about the fidelity of a full 3D model. We have not performed any work using alternative measuring methods, laser-based measurements, imaging of the printed version, or even printing versions cut at the clinical measurement plane. Further work can be performed and is planned using such alternative measuring methods, although for our current purposes, accuracy down to the micron level is probably not required as the vast majority of patients have gross pathology and demonstration of this type of pathology has been demonstrated to be sufficiently appreciable by lay people (unpublished data) using current models. Other work that can also be performed is to cut the models at the clinical (MRI) measurement plane and repeat the measurements at this plane to more accurately represent the measurements obtained on the MR images. However, one potential pitfall of this would be the difficulty in obtaining the exact slice/position on the 3D model given that the cutting would be performed independent of the MRI scan and by estimation of position by gross visualization.

## Conclusion

This paper confirms that 1:1 scale 3D printed models of the brain derived from MRI scans of children with hypoxic ischemic injury maintain adequate fidelity of linear measurements and can therefore be used for medical training, clinical consultation, communication, and medical courtroom evidence.

Further refinements may result in potential improvements to further increase fidelity, which would encourage use of 3D models for detailed surgical planning, simulation, and demonstration.
